# Chronic ankle instability: a meta-analysis and systematic review comparing clinical outcomes of anterior talofibular ligament repair with or without reinforcement of the lower extensor retinaculum

**DOI:** 10.3389/fsurg.2025.1572345

**Published:** 2025-05-19

**Authors:** Haoyang Liu, Hongxu Li, Mengran Shen, Yu Zhou, Bailiang Wang, Jinhui Ma

**Affiliations:** ^1^ Department of Orthopaedic Surgery, Peking University China-Japan Friendship School of Clinical Medicine, Beijing, China; ^2^ Department of Orthopaedic Surgery, Suqian First People's Hospital, Suqian, Jiangsu, China; ^3^ Department of Orthopaedic Surgery, Center for Osteonecrosis and Joint Preserving & Reconstruction, China-Japan Friendship Hospital, Beijing, China

**Keywords:** anterior talofibular ligament (ATFL), meta-analysis, inferior extensor retinaculum, chronic ankle instability, ankle sprains

## Abstract

**Background:**

Chronic ankle instability (CAI) is a prevalent condition often treated with the Broström procedure, sometimes modified by Gould. This study aims to compare the clinical outcomes of patients undergoing the Broström procedure with and without the Gould modification, focusing on the implications for CAI management.

**Methods:**

A comprehensive search was conducted across PubMed, EMBASE, Wiley Library, Science Direct, Europe PMC, and Scopus for studies comparing the Broström procedure with and without the Gould modification. The search spanned from the inception of these databases to October 12, 2024, using specific terms related to ankle instability and ligament repair.

**Results:**

Our meta-analysis revealed that there was no significant difference in AOFAS scores, indicating a weak correlation between AOFAS scores and reinforcement of the Inferior Extensor Retinaculum (IER) [mean difference −1.14 (−2.16, −0.11), *p* = 0.03 I2:0%, *p* = 1.000]. Similarly, the reinforcement of IER showed a low correlation with Karlsson scores [mean difference −0.15 (−2.25, 1.96), *p* = 0.89; I2: 48%, *p* = 0.07]. The results for talar tilt [mean difference −0.11° (−0.37, 0.15), *p* = 0.42; I2:0%, *p* = 0.87] and anterior talar translation [mean difference 0.09 mm (−0.10, 0.29), *p* = 0.34; I2:0%, *p* = 0.91] were similar between the two groups at follow-up. The funnel plots for AOFAS scores, talar tilt, and complications were symmetrical, indicating no publication bias or other biases in the studies.

**Conclusions:**

The findings suggest that for patients with CAI, the Broström procedure with or without the Gould modification yields comparable postoperative functional outcomes. This has significant implications for the surgical management of CAI, potentially simplifying treatment protocols.

**Level of evidence:** Level II, systematic review of Level II studies.

## Introduction

Ankle sprains are one of the most common recurrent injuries in the lower limbs. Additionally, approximately 20% of acute sprain patients develop chronic ankle instability (CAI) (1). When conservative rehabilitation for CAI fails, surgical treatment is required to reconstruct ankle joint mechanical stability. The original direct anatomical repair of the lateral ankle ligaments described by Broström in 1966 remains the most popular surgical procedure (2). Furthermore, in 1980, Gould proposed the idea of further tightening the inferior extensor retinaculum (IER) on the basis of the Broström procedure, and has achieved good clinical results (3–6). Subsequently, with the use and improvement of arthroscopic techniques, arthroscopic treatment of chronic ankle instability (CAI) has gradually become the mainstream surgery, with recent reports of anatomical repair of the lateral ankle ligaments with or without Gould modification (IER reinforcement) showing good results in the majority of cases (7–9). However, studies comparing the Broström procedure with vs. without the Gould modification—whether performed via open surgery or arthroscopically—have consistently reported no significant differences in clinical outcomes for chronic ankle instability (CAI) (10–18).

Another issue that has been observed in Behrens study is that approximately 64% of the inferior extensor retinaculum (IER) has an X-shaped structure, with only the anterior talofibular ligament (ATFL) located near it (19). However, the anterior talofibular ligament is a weak tissue band, and when used to reinforce ATFL repair, it may have minimal impact on improving ankle joint stability. Furthermore, in all cases, the superficial peroneal nerve essentially passes through the IER anatomically, which may lead to an increased risk of complications following the Broström procedure with Gould modification (12).

As there are conflicting conclusions in current literature and a lack of consensus among physicians regarding the performance of the Broström procedure with or without the Gould modification, the purpose of this review is to conduct a meta-analysis of the current literature and compare patient-reported outcome measures (PROM) in patients undergoing ankle ligament repair (with or without ankle bony reconstruction). We hypothesize that there is no significant clinical difference between patients undergoing the Broström procedure with the Gould modification and those without it.

## Methods

This study follows the recommendation by the Preferred Reporting Items for Systematic Reviews and Meta-Analyses. PROSPERO Registration Number: CRD420251024846.

We conducted a search in PubMed, EMBASE, Wiley Library, Science Direct, Europe PMC, and Scopus, to retrieve all publications comparing the outcomes of patients undergoing the Broström procedure with or without the Gould modification. The search terms included: “(Brostrom OR Gould OR Inferior Extensor Retinaculum Augmentation OR Anterior Talofibular Ligament Repair OR inferior extensor retinaculum) AND (lateral ankle OR ankle instability)”, covering the period from the establishment of the databases to October 12, 2024. Two independent reviewers excluded review articles, case reports, case series, cadaveric studies, basic science research, animal studies, studies with incomplete reporting methods, and studies that did not directly compare the Broström procedure with the Gould modification to the Broström procedure without the Gould modification. At each stage of the selection process, any discrepancies in study selection were discussed and resolved by the two independent reviewers. The characteristics of the included studies were synthesized and described in the methods section. In cases where there were discrepancies in the assessment of risk of bias, the two reviewers discussed and resolved the differences. Table 1 describes the various rehabilitation regimens included in the selected studies. Ankle function scores (AOFAS and Karlsson scores), measurements of talar tilt, anterior talar translation were used to estimate the mean difference in outcomes, and odds ratios (OR) for complications.

**Table 1:** Baseline characteristics of the included studies.

Author	Study design	Inclusion criteria	Technique	Rehabilitation	Number of anchor (s)	Talar tilt (mm)	Anterior drawer test (mm）	Age	Male	BMI	Mean follow-up (months)	NOS	Follow-up, months
Jeong et al. (2014) (11)	Prospective cohort	CLAI, operated between February 2011 and October 2012	Arthroscopic BP + IERvs. Arthro-scopicBP	• Non-weight-bearing was maintained for 6 weeks after surgery. • Short leg cast immobilization was maintained for 4 weeks after surgery. • Ankle ROM exercises were allowed for 2 weeks with protection of ankle orthosis. • Six weeks after surgery begins partial weight-bearing,proprioception, and peroneal muscle strengthening exercise. • Light exercises began after 3 months and gradually returned to their normal sports activities	1 vs. 1	Arthroscopic BP + IER 5.0 ± 2. Arthroscopic BP 4.9 ± 0.9	(Talar anterior translation) Arthroscopic BP + IER 4.6 ± 1.9 Arthroscopic BP 4.9 ± 0.8	26.2	51.6	N/A	19.5 vs. 18.9	7	19
Yeo et al. (2016) (17)	Randomized clinical trial	CLAI, operated between August 2012 and July 2014	Arthroscopic BP vs. Open BP + IER	• All patients were placed in a well-padded posterior splint with the foot in slight dorsiflexion and kept non-weight-bearing until 2 weeks. • Short leg walking cast for next 2 weeks and protected progressive weight-bearing was then allowed. • During week 4–6, half-removed cast or splint was applied and started on gentle active assisted ROM of the ankle and peroneal strengthening exercise. • Eight weeks postoperatively the patient began running and functional activities. • Cutting and sport-specific drills were started by week 12	1 vs. 1	Arthroscopic BP 3.9 ± 1.5 Open BP + IER 3.8 ± 3.6	Arthroscopic BP 6.7 ± 1.3 Open BP + IER 6.8 ± 2.1	34.8	39.6	N/A	12 vs. 12	7	12
Araoye et al. (2017) (10)	Retrospective cohort	CLAI, operated between 2006 and 2016	Arthroscopic BP + IER vs. Arthroscopic BP	N/A	Suture anchor vs. Direct suture	N/A	N/A	40	27.8	31.9	11.8	7	11.5
Li et al. (2017) (14)	Prospective cohort	CLAI, operated between January 2012 and August 2014	Arthroscopic BP vs. Open BP + IER	• Isometric contraction from the day after surgery. • The ankle was immobilized in a neutral position by short leg cast • Two weeks after the surgery, the cast was changed to an ankle brace and passive ROM was encouraged • Weight-bearing was permitted after 4 weeks	1 or 2 vs. 1 or 2	N/A	N/A	29.3	78.3	23.7	39.7 vs. 35.5	7	24
Gang et al. (2020) (23)	Retrospective cohort	CLAI, operated between January 2014 and January 2017	Arthroscopic BP vs. Open BP + IER	• Short leg cast was applied after surgery and isometric contraction was trained. • After 2 weeks, short leg cast was removed and replaced by functional exercise brace, and partial weight-bearing was started. (active and passive ROM exercise was initiated) • Six weeks after surgery, the patient began balance training, endurance training, and transition to full weight-bearing.	1 vs. 1	Arthroscopic BP 3.3 ± 0.8 Open BP + IER 3.1 ± 0.4	(Anterior displacement of Talus) Arthroscopic BP 3.2 ± 0.4 Open BP + IER 3.3 ± 0.5	38.4	70.1	N/A	26 vs. 26	9	12
Xu et al. (2020) (16)	Retrospective cohort	CLAI accompanied by OLT, operated between May 2015 and May 2017	Arthroscopic BP vs. Open BP + IER	• The ankle was protected by an ankle brace for 6 weeks. • Isometric contraction of muscle groups was allowed from the day after surgery • Passive and active ROM was allowed from the 7th day after surgery under ankle brace. • Partial weight-bearing began from the 5th week after surgery and full weight-bearing began from 7th week after surgery • The patient could return to high-impact physical activities for 6 months after surgery	1 or 2 vs. 1 or 2	N/A	N/A	34.8	73.1	23.7	36.5 vs. 39.1	9	24
Zeng et al. (2020) (18)	Retrospective cohort	CLAI, operated between January 2013 and June 2015	Arthroscopic BP vs. open BP + IER	• The short leg cast was applied until 2 weeks after surgery. • After 2 weeks, short leg cast was removed. Flexion and extension of hip and knee were encouraged • At 3–6 weeks after surgery, patients began walking in a boot, and strengthening exercises of the whole lower extremity. • The flexion and extension of the ankle joint were passively performed. • At 6–12 weeks after surgery, balance training and full weight-bearing were performed	1 vs. 1	Arthroscopic BP 2.7 ± 1.2 Open BP + IER 2.4 ± 1.3	Arthroscopic BP 3.3 ± 1.3 Open BP + IER 2.8 ± 1.1	29.7	81.5	N/A	36 vs. 36	9	36
Lee et al. (2021) (13)	Retrospective cohort	CLAI, operated between January 2016 and December 2018	Arthroscopic BP + IERvs. Arthroscopic BP	• A compression bandage and a posterior plaster splint were applied with the ankle in a neutral position and maintained for 2–3 days after the surgery. • A removable walking boot was used for 6 weeks. • Full weightbearing and active range of motion exercises were permitted from 2 weeks after the surgery.	1 vs. 1	N/A	Arthroscopic BP + IER 5.4 ± 2.2 Arthroscopic BP 5.3 ± 2.7	35.5	16	28.5	17 vs. 17	9	32.6
Samejima et al. (2021) (15)	Retrospective cohort	CLAI, operated between January 2017 and December 2019	Arthroscopic BP + IERvs. Arthroscopic BP	• The splint was not used after surgery, and weight-bearing was permitted from the day after surgery • The restriction on plantar flexion was lifted after 4 weeks • Sport-specific drills, proprioceptive training, and jogging (<8 km/h) began 2 weeks after surgery.	1 vs. 1	N/A	N/A	38.5	25	N/A	24 vs. 24	9	12
Jo et al. (2021) (12)	Prospective cohort	CLAI, operated between May 2018 and August 2019	Arthroscopic BP + IERvs. Arthroscopic BP	• A non-weight-bearing splint was applied and worn for 2 weeks, • After 2 weeks, short leg cast was removed. Flexion and extension of hip and knee were encouraged • At 4 weeks postoperatively, patients were converted to the use of an ankle stirrup brace, and postoperative rehabilitation exercises comprising active range of motion, muscle strengthening, Achilles tendon stretching, and proprioception exercises were initiated. • At 8 weeks postoperatively, the patient was weaned off the ankle stirrup brace.	1 vs. 1	Arthroscopic BP + IER 5.0 ± 3.1 Arthroscopic BP 4.5 ± 3.1	Arthroscopic BP + IER 5.9 ± 1.7 Arthroscopic BP 5.8 ± 1.3	30	41	25.7	15 vs. 15	9	12

CLAI, chronic lateral ankle instability; BP, Broström procedure; IER, inferior extensor retinaculum augmentation; OLT, osteochondral lesion of the talus; NOS, Newcastle-Ottawa scale; ROM, range of motion; BMI, body mass index.

The AOFAS score can be used to grade the level of ankle function (20). A score of 100 points is given if the patient is free of pain, has a wide range of sagittal and hindfoot motion, with no instability in the ankle or hindfoot, good alignment, able to walk more than six blocks, walk on any surface, no significant limping, and no restriction in daily or recreational activities, without the need for walking aids. Fifty points are assigned to function, 40 points to pain, and 10 points to alignment. The Karlsson-Peterson score, on the other hand, assesses functional outcomes based on joint instability, pain, swelling, and stiffness, with a maximum score of 100 (21). We conducted a meta-analysis using Review Manager 5.3. A restricted maximum likelihood (REML) random-effects model was applied to calculate the mean differences (MDs) and 95% confidence intervals (CIs) for AOFAS score, Karlsson score, talar tilt, and anterior drawer test between the intervention and control groups. Additionally, a DerSimonian-Laird random-effects model was used to estimate the odds ratio (OR) and 95% CIs for complications. To assess interstudy heterogeneity, we performed I^2^ statistics and Cochran's *Q*-test, with I^2^ > 50% and *p* < 0.10 indicating substantial heterogeneity. Furthermore, we conducted a meta-regression analysis with age, male proportion, and BMI as covariates. Sensitivity analysis was performed by excluding studies with potential selection bias to evaluate the robustness of the results.

## Results

**Figure 1:**
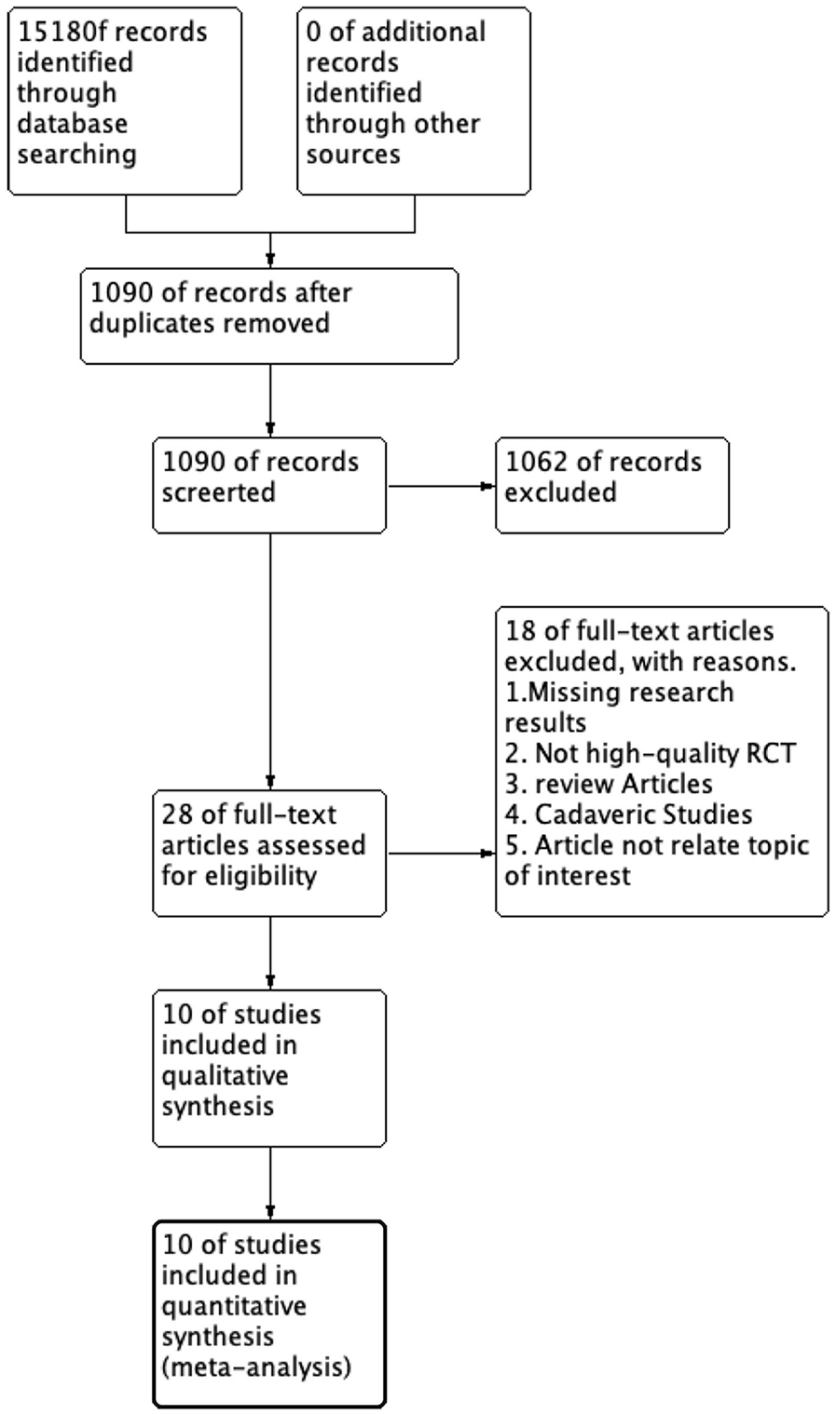
Preferred reporting items for systematic reviews and meta-analyses diagram on article selection for systematic review.

**Figure 2:**
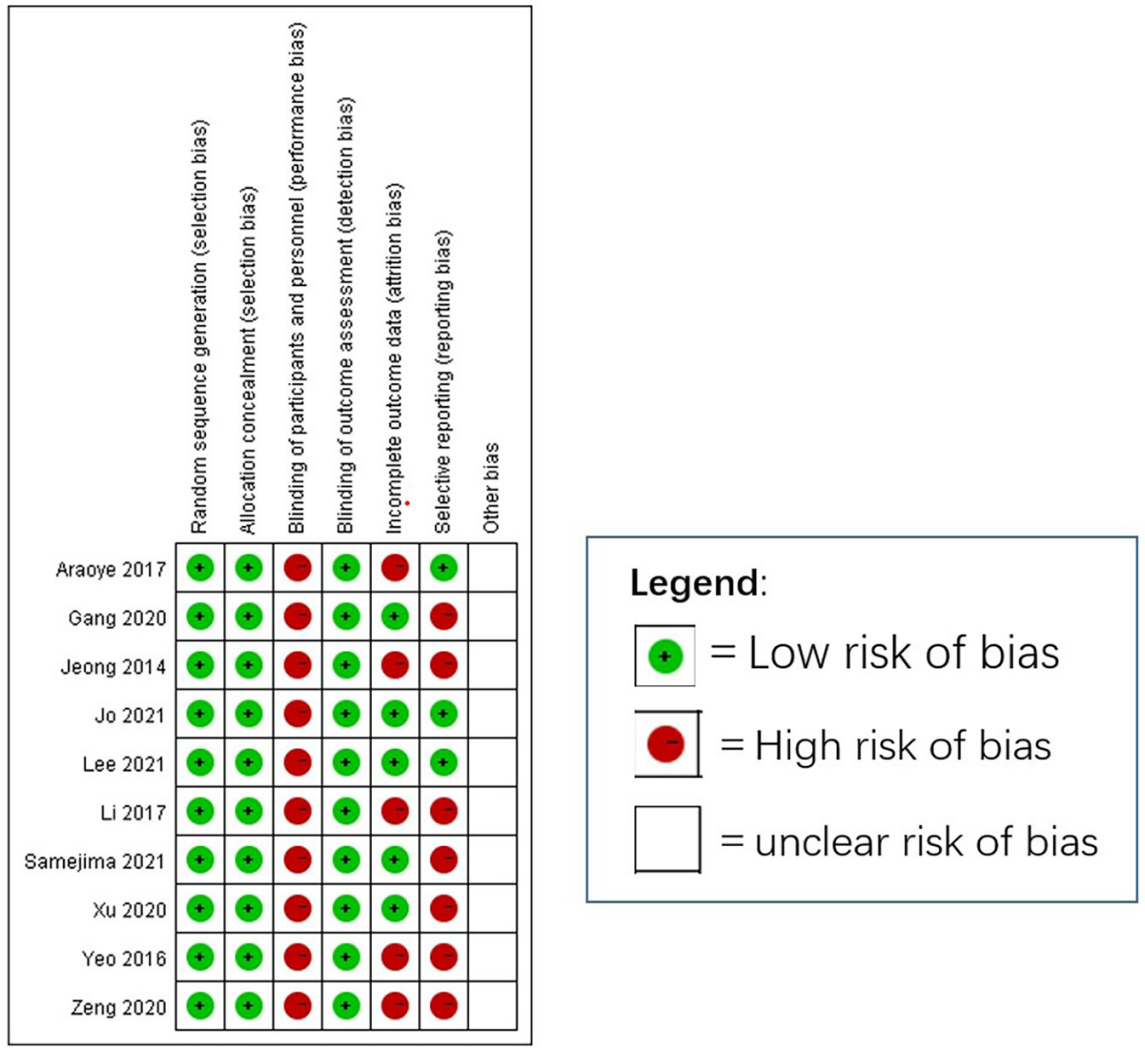
Summary of quality assessment of randomised trials.

We initially identified 1,518 studies from the databases. After removing duplicates, 1,090 studies remained. Based on inclusion/exclusion criteria (e.g., randomized controlled trials, observational studies) and excluding non-comparative research (e.g., reviews, case reports, animal studies), we screened 28 studies for eligibility. Studies were excluded if they did not directly compare the Broström procedure with the Gould modification to the Broström procedure without the Gould modification. Ultimately, 10 studies (*n* = 635 patients) were included. The mean follow-up period was 19.5months (11.5–36 months) across all included studies. We summarized the selection process for each study in Figure 1 and presented the 10 studies in Table 1. Figure 2 (Cochrane risk of bias tool for randomized studies) provided a summary of the risk of bias assessment, with a *κ* score of 0.7, indicating substantial agreement. We analyzed four outcomes: ankle function scores (AOFAS and Karlsson scores), talar tilt, and anterior talar translation for the included studies.

**Figure 3:**
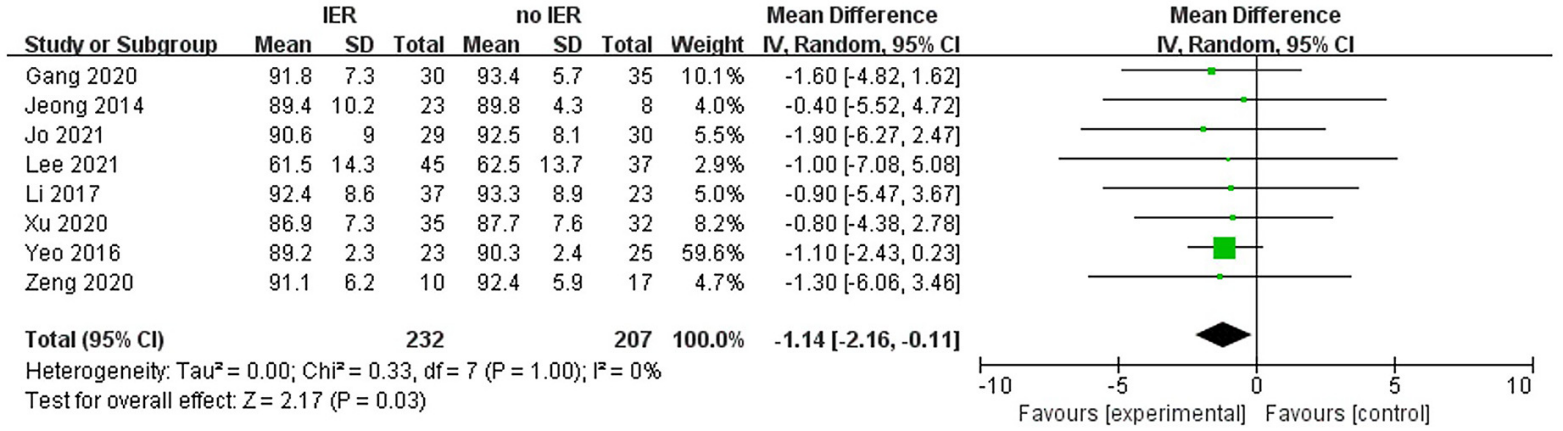
Forest plot comparing postoperative AOFAS, American orthopaedic foot & ankle society and between IER and noIER groups.

**Figure 4:**
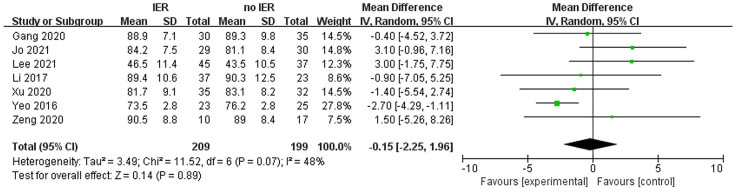
Forest plot comparing postoperative Karlsson score between IER and noIER groups.

**Figure 5:**
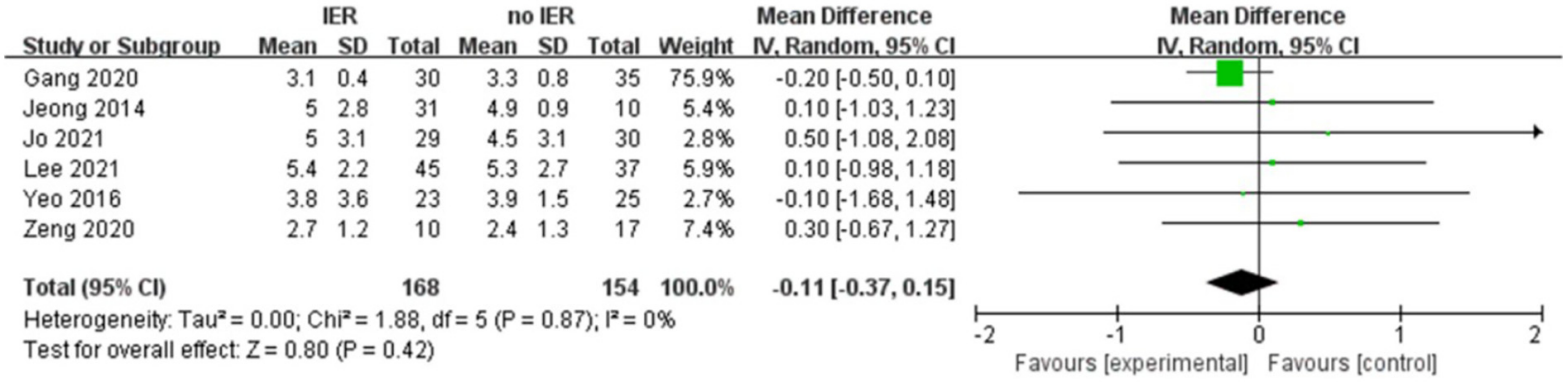
Forest plot comparing postoperative talar tilt between IER and no IER groups.

**Figure 6:**
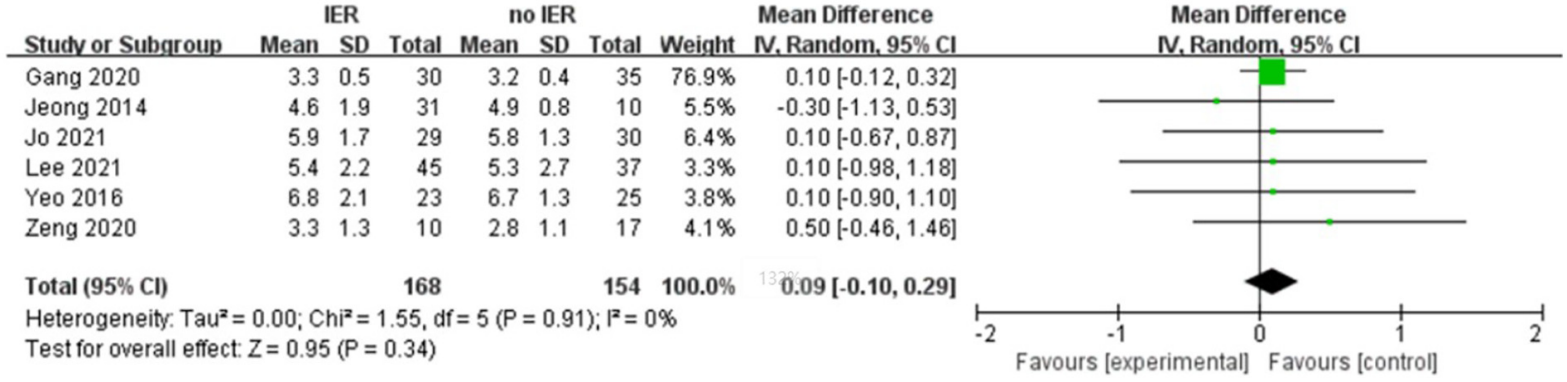
Forest plot 8comparing postoperative anterior displacement between IER and no IER groups.

**Figure 7:**
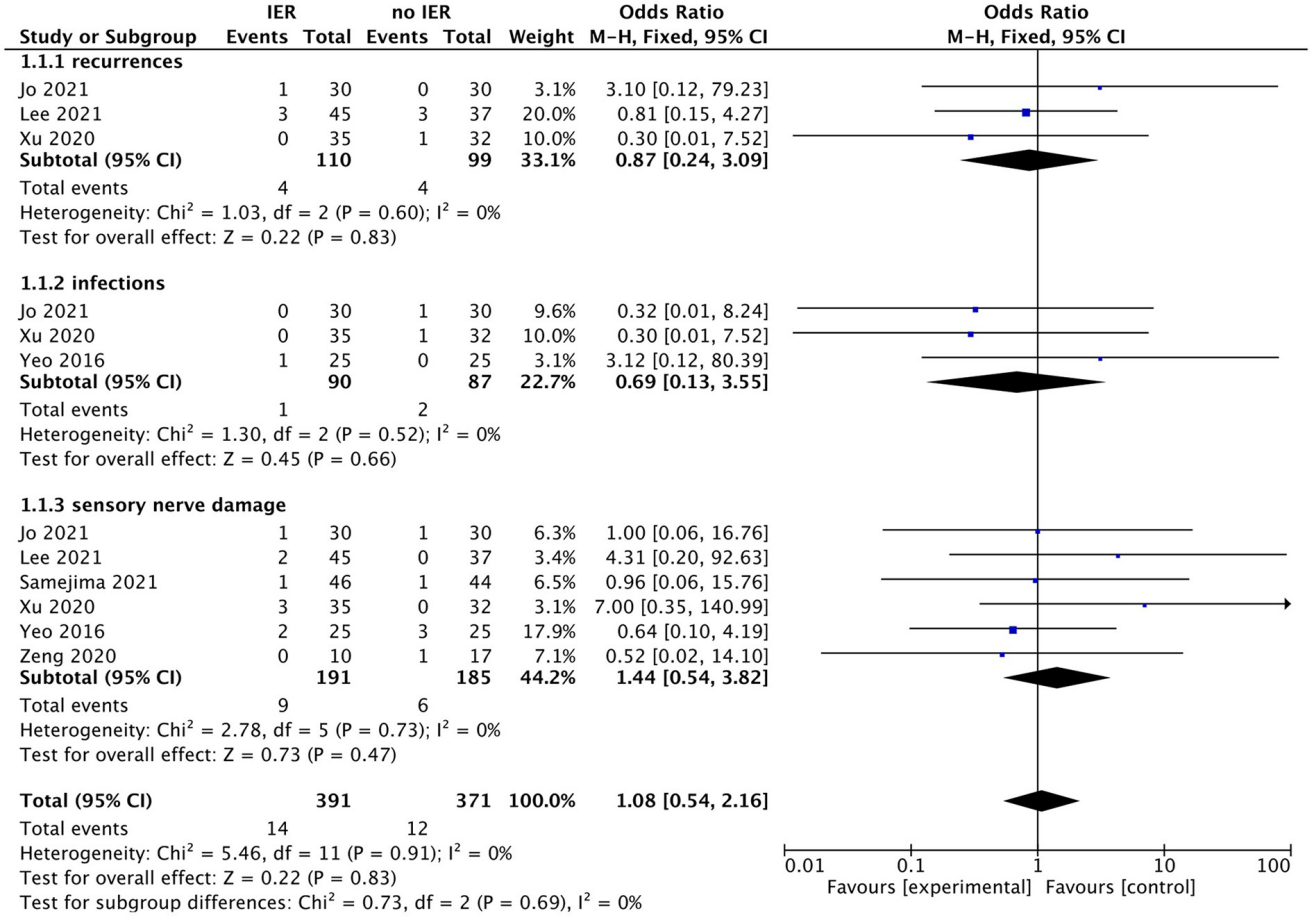
Forest plot comparing postoperative complications between IER groups and no IER groups.

**Figure 8:**
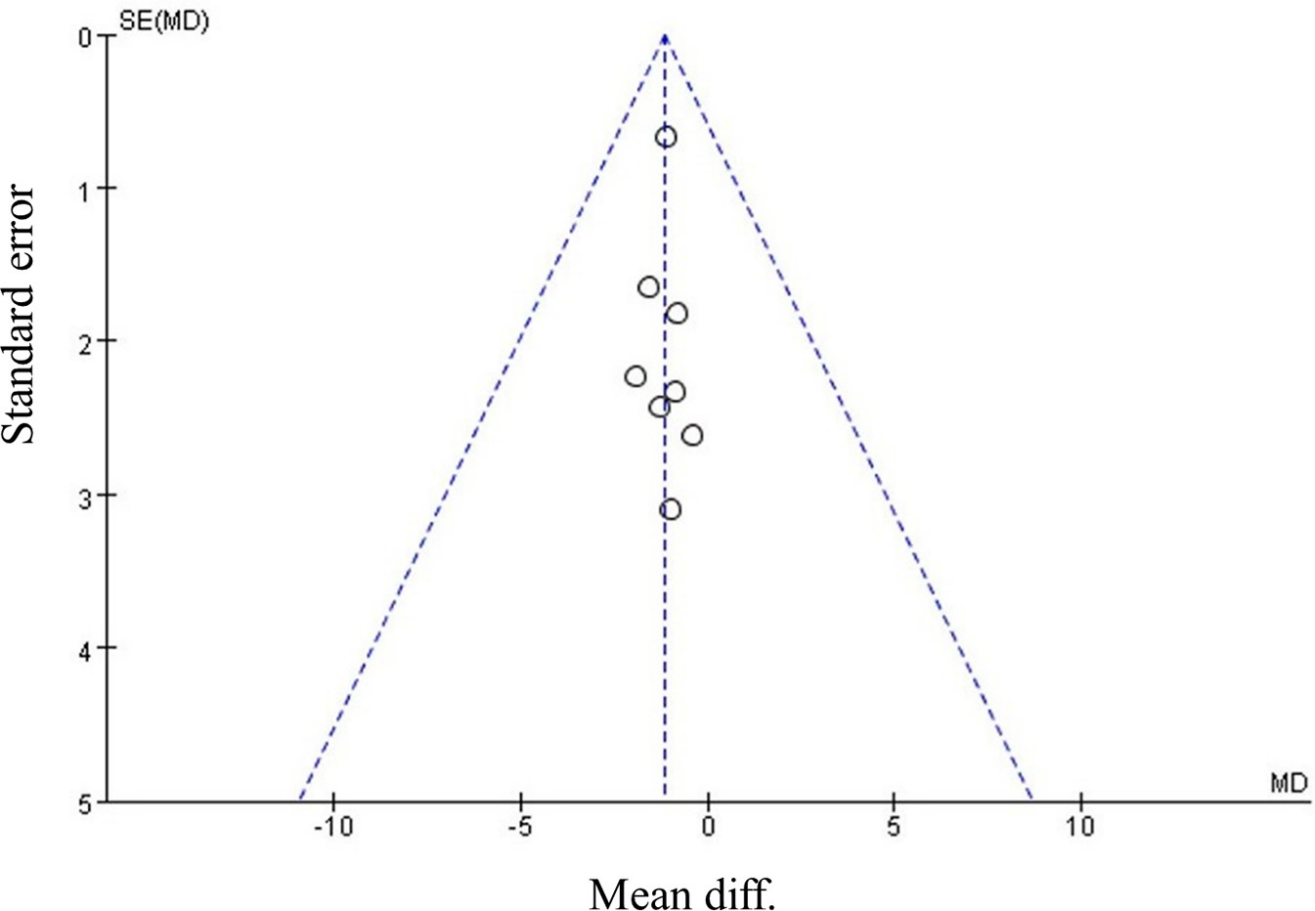
Funnel plot comparing postoperative AOFAS, American orthopaedic foot & ankle society between IER and noIER groups.

Our meta-analysis revealed that there was no significant difference in AOFAS scores, indicating a weak correlation between AOFAS scores and reinforcement of the Inferior Extensor Retinaculum (IER) [mean difference −1.14 (−2.16, −0.11), *p* = 0.03 I^2^:0%, *p* = 1.000] (Figure 3). Similarly, the reinforcement of IER showed a low correlation with Karlsson scores [mean difference −0.15 (−2.25, 1.96), *p* = 0.89; I^2^: 48%, *p* = 0.07] (Figure 4). The results for talar tilt [mean difference −0.11° (−0.37, 0.15), *p* = 0.42; I^2^:0%, *p* = 0.87] (Figure 5) and anterior talar translation [mean difference 0.09 mm (−0.10, 0.29), *p* = 0.34; I^2^:0%, *p* = 0.91] were similar between the two groups at follow-up (Figure 6). No statistically significant differences were observed between arthroscopic non-IER augmentation and open IER augmentation in postoperative AOFAS scores [mean difference −1.11, 95% CI (−1.80, −0.01), *p* = 0.66; I^2^ = 0%, *p* = 1.000] or Karlsson scores [mean difference −2.00, 95% CI (−2.23, −0.45), *p* = 0.56; I^2^ = 2.63%, *p* < 0.001]. While minor heterogeneity was detected in Karlsson scores for the open IER group (I^2^ = 2.62%), its clinical impact was negligible. Subgroup analyses revealed no statistically significant differences in specific complications between the Broström procedure with and without IER reinforcement. The pooled odds ratios (ORs) were as follows: Recurrence: OR = 0.87 [95% CI (0.24, 3.09), *p* = 0.83; I^2^ = 0%]. Infections: OR = 0.69 [95% CI (0.13, 3.55), *p* = 0.66; I^2^ = 0%]. Sensory nerve damage: OR = 1.44 [95% CI (0.54, 3.82), *p* = 0.47; I^2^ = 0%]. The homogeneity (I^2^ < 15%) across all subgroups suggests minimal variability in complication reporting between studies (Figure 7). The funnel plots for AOFAS scores (Figure 8), talar tilt, and complications were symmetrical, indicating no publication bias or other biases in the studies.

## Discussion

The results of this meta-analysis indicate that there were no significant differences in postoperative AOFAS and Karlsson scores, as well as talar tilt angle and anterior translation, when comparing patients who underwent the Broström procedure with the Gould modification and those who did not undergo the Gould modification. These findings are also supported by the assessment of other outcome scores in our systematic review, such as the JSSF scale and SAFE-Q (15). Most ankle assessment systems consist of multiple components, including functional parameters reported by the patient (such as the ability to participate in daily and athletic activities), patient-reported parameters related to quality of life (such as pain), other forms of psychological or psychosocial measures, and clinical assessments such as range of motion and strength measurements. Although differences exist in the arthroscopic surgery, open surgery, and rehabilitation protocols among the included studies, the low heterogeneity in the summary results and the similar conclusions drawn indicate consistency. Araoye et al. observed no subjective instability in postoperative patients in both groups (with or without Gould modification) and reported high satisfaction levels (10). Additionally, in clinical trials where random allocation was not always feasible, the trial results were not affected. Jeong et al. also included an analysis of 10 patients with complications during follow-up, such as concomitant bone and cartilage injuries, ankle joint fractures, and subtalar instability (11). The clinical measurements and patient satisfaction did not show significant differences among the groups, as evidenced by the low heterogeneity (I^2^ = 0). While our analysis confirmed comparable rates of sensory nerve damage, infections, and recurrence between groups, the impact of these complications on patient-reported outcomes (PROs) such as quality of life, pain perception, or functional recovery remains unexplored. This limitation stems from inconsistent reporting of PROs in the included studies, which predominantly focused on clinician-assessed metrics (e.g., AOFAS scores). Moreover, the included studies had relatively shorter follow-up durations (with a mean of 11.5–36 months), which may be insufficient to capture long-term recurrence events. Future studies should prioritize integrating validated PRO measures (e.g., SF-36, FAAM) alongside traditional clinical outcomes to holistically evaluate the patient experience. Some studies indicate that the Gould modification may not provide anatomical repair, can increase surgical time and complexity, and may lead to complications such as superficial peroneal nerve or sural nerve neuritis with a reported complication rate of approximately 14% (8, 22). However, when the lateral ligament injury of the ankle is severe and complete anatomical reduction is not achievable, reinforcement of the Inferior Extensor Retinaculum (IER) might be used to enhance ankle stability. It is suggested in the research to add a grading system for the severity of ATFL (Anterior Talofibular Ligament) injuries: Grade 0, ligament is normal and continuous, no tear, normal thickness, and taut between the lateral malleolus and the talus neck; Grade 1, ligament is stretched, no tear, normal thickness, but reduced tension on palpation; Grade 2, partial avulsion of the fibula or talus involving the ATFL (including fibrous tissue) with normal thickness, but reduced tension on palpation; Grade 3, thinning of the ATFL, lack of mechanical resistance on palpation, with or without scar tissue; Grade 4, scar tissue replacing the ATFL. This grading system can help minimize selection bias and provide more precise understanding of the outcomes associated with different degrees of ATFL injuries in different surgical procedures.

## Limitations

While the sample size (10 studies) may limit generalizability, pooled outcomes showed low heterogeneity, supporting result consistency. Most studies were retrospective, but sensitivity analyses confirmed robustness. Variability in surgical techniques and unreported preoperative status were noted; however, subgroup analyses (e.g., open vs. arthroscopic) revealed no significant differences. Future RCTs with standardized ATFL grading and surgical protocols could refine these findings.

## Conclusions

The findings suggest that for patients with CAI, the Broström procedure with or without the Gould modification yields comparable postoperative functional outcomes. This has significant implications for the surgical management of CAI, potentially simplifying treatment protocols.
